# Alterations in nuclear structure promote lupus autoimmunity in a mouse model

**DOI:** 10.1242/dmm.024851

**Published:** 2016-08-01

**Authors:** Namrata Singh, Duncan B. Johnstone, Kayla A. Martin, Italo Tempera, Mariana J. Kaplan, Michael F. Denny

**Affiliations:** 1 Internal Medicine, University of Iowa, Iowa City, IA 52242, USA; 2 Section of Nephrology, Internal Medicine, Temple University School of Medicine, Philadelphia, PA 19140, USA; 3 Department of Microbiology/Immunology, Fels Institute for Cancer Research, Temple University, Philadelphia, PA 19140, USA; 4 Systemic Autoimmunity Branch, National Institute of Arthritis and Musculoskeletal and Skin Diseases, National Institutes of Health, Bethesda, MD 20892, USA; 5 Section of Rheumatology, Temple University School of Medicine, Philadelphia, PA 19140, USA

**Keywords:** Nucleus, Chromatin, Histone modifications, Calreticulin, Lamina, Autoantibody

## Abstract

Systemic lupus erythematosus (SLE) is an autoimmune disorder characterized by the development of autoantibodies that recognize components of the cell nucleus. The vast majority of lupus research has focused on either the contributions of immune cell dysfunction or the genetics of the disease. Because granulocytes isolated from human SLE patients had alterations in neutrophil nuclear morphology that resembled the Pelger–Huet anomaly, and had prominent mis-splicing of mRNA encoding the nuclear membrane protein lamin B receptor (LBR), consistent with their Pelger–Huet-like nuclear morphology, we used a novel mouse model system to test the hypothesis that a disruption in the structure of the nucleus itself also contributes to the development of lupus autoimmunity. The lupus-prone mouse strain New Zealand White (NZW) was crossed with c57Bl/6 mice harboring a heterozygous autosomal dominant mutation in *Lbr* (B6.Lbr^*ic*/+^), and the (NZW×B6.Lbr*^ic^*)F_1_ offspring were evaluated for induction of lupus autoimmunity. Only female (NZW×B6.Lbr*^ic^*)F_1_ mice developed lupus autoimmunity, which included splenomegaly, kidney damage and autoantibodies. Kidney damage was accompanied by immune complex deposition, and perivascular and tubule infiltration of mononuclear cells. The titers of anti-chromatin antibodies exceeded those of aged female MRL-Fas*^lpr^* mice, and were predominantly of the IgG2 subclasses. The anti-nuclear antibody staining profile of female (NZW×B6.Lbr*^ic^*)F_1_ sera was complex, and consisted of an anti-nuclear membrane reactivity that colocalized with the A-type lamina, in combination with a homogeneous pattern that was related to the recognition of histones with covalent modifications that are associated with gene activation. An anti-neutrophil IgM recognizing calreticulin, but not myeloperoxidase (MPO) or proteinase 3 (PR3), was also identified. Thus, alterations in nuclear structure contribute to lupus autoimmunity when expressed in the context of a lupus-prone genetic background, suggesting a mechanism for the development of lupus autoimmunity in genetically predisposed individuals that is induced by the disruption of nuclear architecture.

## Abstract (precis)

**Summary:** Combining a disruption in nuclear structure with a lupus-prone genetic background induces autoimmunity, suggesting that nuclear alterations trigger the disease in genetically susceptible individuals.

## INTRODUCTION

Systemic lupus erythematosus (SLE) is regarded as a failure of the immune system to maintain tolerance to self-antigens (Tsokos, 2011; Choi et al., 2012). Despite steady advances identifying the importance of the immune system and inflammatory mediators (Deng and Tsao, 2010; Lopez de Padilla and Niewold, 2016), including type I interferons (Elkon and Stone, 2011; Elkon and Wiedeman, 2012), in mediating the progression and severity of the disease, the underlying mechanisms driving the initiation and development of lupus autoimmunity remain unresolved. SLE is characterized by the presence of autoantibodies recognizing components of the cell nucleus, and is thought to originate from loss of tolerance to chromatin that spreads to other components, including histones and DNA. Although immune system dysfunction and the associated loss of tolerance to nuclear autoantigens remains an area of intense investigation, the potential role for alterations in the structure of the nucleus itself remains unexamined.

Alterations in nuclear structure have been reported previously in cells isolated from SLE patients; however, their presence is commonly attributed to a consequence of disease activity rather than a contributor to disease incidence. Cell aspirates from the bone marrow of individuals with lupus have a variety of abnormalities (Voulgarelis et al., 2006; Oka et al., 2008; Papageorgiou et al., 2013), and alterations in nuclear morphology secondary to SLE is an exclusion in the diagnosis of myelodysplastic disorders. Similarly, alterations in the nuclear morphology of neutrophils in systemic circulation have been identified (Hacbarth and Kajdacsy-Balla, 1986; Bennett et al., 2003; Denny et al., 2010; Singh et al., 2014). Given the nature of SLE as an autoimmune disorder that targets the cell nucleus, an alternative model for induction of the disease is that alterations in nuclear structure promote the development of an immune response that initially targets aberrant nuclei, which then spreads to include additional components of the nucleus.

The nucleus is stabilized by a network of proteins contained within the lamina (Gotzmann and Foisner, 1999; Holaska et al., 2002), and components of the nuclear lamina have been identified as targets of autoimmunity (Reeves et al., 1987; Courvalin et al., 1990; Chou and Reeves, 1992; Senecal et al., 1999). Lamins A and C are anchored to the nuclear membrane by scaffold proteins, including the lamina-associated polypeptides, whereas B-type lamins are anchored by lamin B receptor (LBR) (Gotzmann and Foisner, 1999; Holmer and Worman, 2001; Worman, 2005; Gruenbaum and Medalia, 2015). LBR spans the inner nuclear membrane and has a DNA-binding region that associates with heterochromatin to maintain its localization at the membrane margins (Smith and Blobel, 1993; Ye and Worman, 1994; Pyrpasopoulou et al., 1996; Polioudaki et al., 2001; Makatsori et al., 2004; Solovei et al., 2013). Impaired expression of LBR results in autosomal dominant disruptions in nuclear structure that include an alteration in neutrophil nuclear morphology called the Pelger-Huet anomaly (Hoffmann et al., 2002; Worman, 2005). In mice, *Lbr* is within the Sle1 lupus susceptibility interval on chromosome 1 derived from the New Zealand White (NZW) strain (Morel et al., 1997, 2001; Mohan et al., 1998; Shultz et al., 2003). Given the role of Lbr in stabilizing nuclear structure and its ability to bind chromatin, it is conceivable that disruptions in the *Lbr* gene might contribute to the development of autoimmunity, particularly in genetically predisposed individuals. A mouse model was developed in which a heterozygous defect in *Lbr* mRNA splicing was expressed in the context of a hemizygous lupus-prone NZW genetic background. Disruption of *Lbr* promoted the development of lupus autoimmunity in female offspring, consistent with nuclear alterations contributing to the disease when expressed in a susceptible genetic background.

## RESULTS

### Granulocytes from human SLE patients have defects in *LBR* mRNA splicing

**Fig. 1.**
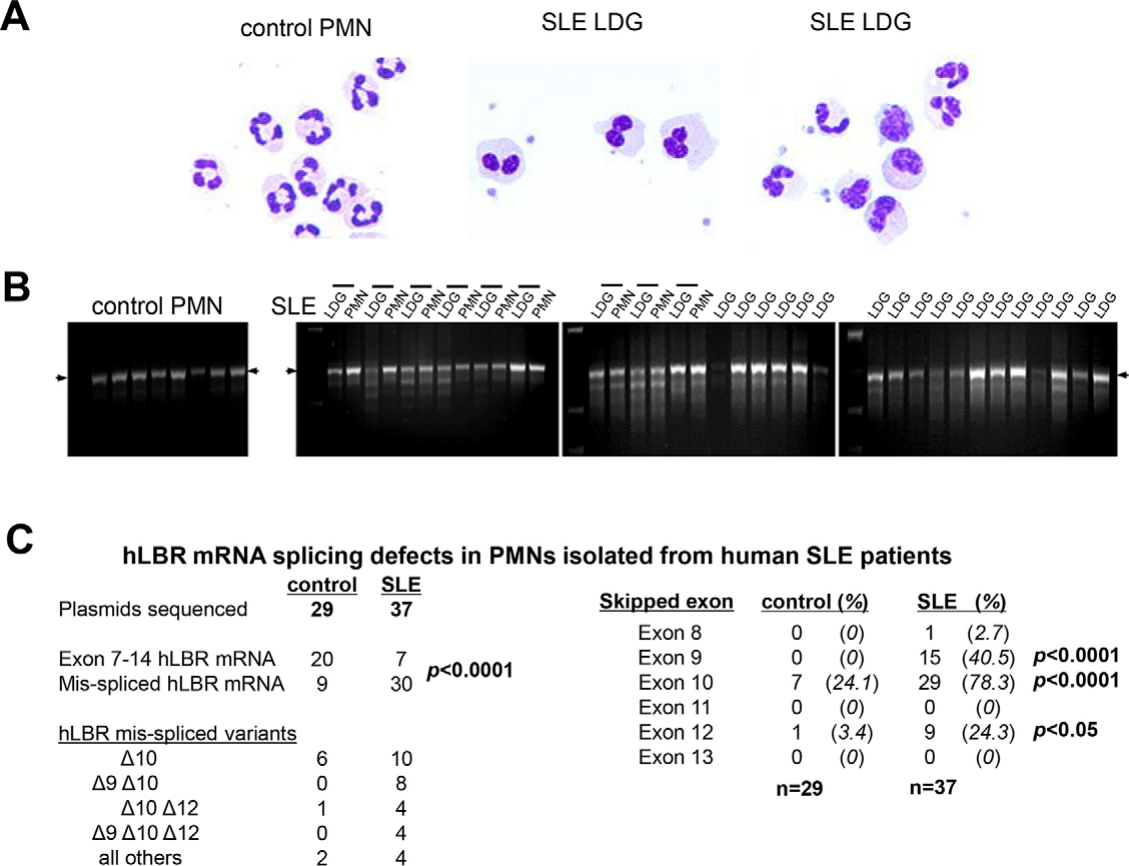
**LBR splicing defects in PMNs isolated from SLE patients.** (A) Differential staining of normal density neutrophils isolated from a healthy individual, and the LDGs from two SLE patients, demonstrates the Pelger–Huet-like nuclear morphology of SLE neutrophils. Image magnification, 400×. (B) PCR amplification of exons 7 to 14 of human LBR cDNA prepared from control PMNs, SLE normal density PMNs and LDGs. The horizontal lines above the sample lanes reflect autologous pairs of LDGs and normal density PMNs isolated in parallel. The arrow indicates the position of properly spliced human LBR. (C) Subcloning and sequencing of human LBR cDNA amplicons verified the mis-splicing of mRNA, and identified extensive skipping of LBR exon 10, both alone and in combination with exons 9 and/or 12. Statistical significance assessed by Fisher's exact test, one-tailed.

Patients with SLE have neutrophils with lobulated or ovoid nuclei (Hacbarth and Kajdacsy-Balla, 1986; Bennett et al., 2003; Voulgarelis et al., 2006; Denny et al., 2010; Singh et al., 2014). This alteration in neutrophil nuclear morphology in SLE resembles the Pelger–Huet anomaly (Fig. 1A), an autosomal dominant genetic disorder that results in nuclear alterations due to impaired expression of the nuclear membrane protein LBR (Hoffmann et al., 2002; Shultz et al., 2003; Olins et al., 2008). Low-density granulocytes (LDGs) and polymorphonuclear cells (PMNs) isolated from SLE patient samples were examined for defects in *LBR* mRNA expression. Defective *LBR* mRNA splicing in SLE neutrophils was apparent in the cDNA amplicons between exons 7 to 14 (Fig. 1B), displaying a laddering pattern consistent with exon skipping. The PCR products were subcloned and sequenced to determine which *LBR* exons were prone to mis-splicing in PMNs isolated from SLE patients. Of the 29 *LBR* plasmids sequenced from five healthy controls, 20 were properly spliced. In contrast, only 7 of 37 *LBR* subclones obtained from four pairs of LDGs and PMNs isolated from SLE patients encoded intact *LBR* mRNA. Because the incidence of mis-spliced *LBR* amplicons was similar between LDGs and PMNs, these samples were analyzed together for splicing errors. *LBR* exon 10 was skipped in 29 mis-spliced cDNA amplicons from SLE patients (Fig. 1C), and frequently occurred in combination with skipping of exon 9 and/or exon 12. Thus, the splicing of the nuclear envelope protein LBR is disrupted in PMNs from SLE patients, consistent with their Pelger–Huet-like nuclear morphology; these findings support a model in which alterations in nuclear structure are present in SLE patients.

### Nuclear alterations promote the development of lupus autoimmunity in a lupus-prone background

**Fig. 2.**
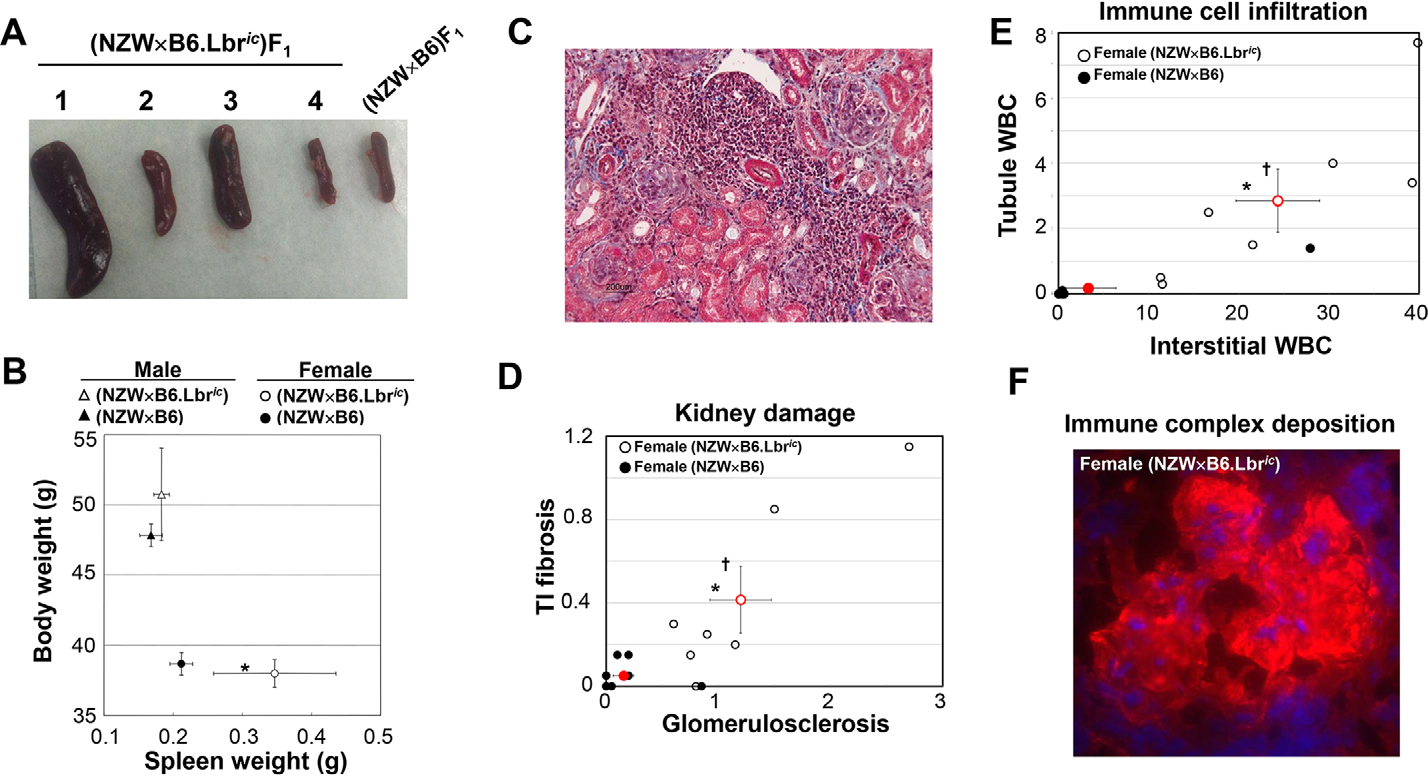
**Development of splenomegaly and kidney damage in female (NZW×B6.Lbr*^ic^*)F_1_ mice.** (A) Intact spleens isolated from four female (NZW×B6.Lbr*^ic^*)F_1_ mice and one female (NZW×B6)F_1_ control. The spleens of three female (NZW×B6.Lbr*^ic^*)F_1_ mice are enlarged, whereas the size of the fourth is similar to that of the control. (B) Mean body weight (*y*-axis) and splenic mass (*x*-axis) in male (triangles) and female (circles) mice of the NZW×B6 (filled symbols) and NZW×B6.Lbr*^ic^* (open symbols) genotype. Error bars represent s.e.m. Asterisk (*) indicates significant difference spleen weight in female (NZW×B6.Lbr*^ic^*)F_1_ mice relative to female (NZW×B6)F_1_ littermates (Student's *t*-test, two-tailed, *P*<0.05). (C) Masson's Trichrome-stained kidney section representative of the female (NZW×B6.Lbr*^ic^*)F_1_ mice with the most severe kidney damage and perivascular cellular infiltration. Image magnification, 200×. (D) Histological evaluation of Masson's Trichrome-stained sections for kidney damage. Female (NZW×B6)F_1_ (solid circles) and (NZW×B6.Lbr*^ic^*)F_1_ (open circles) were assessed for tubulointerstitial fibrosis (*y*-axis) and glomerulosclerosis (*x*-axis). Red symbols represent the mean and s.e.m. of the corresponding group. Asterisk (*) indicates significant difference in glomerulosclerosis, dagger (†) indicates significant difference in tubulointerstitial fibrosis, Mann–Whitney test, one-tailed. (E) Histological evaluation of Masson's Trichrome-stained sections for cellular infiltration. Female (NZW×B6)F_1_ (solid circles) and (NZW×B6.Lbr*^ic^*)F_1_ (open circles) were assessed for tubule white blood cell infiltration (*y*-axis) and interstitial infiltration (*x*-axis). Red symbols represent the mean and s.e.m. of corresponding group. Asterisk (*) indicates significant difference in interstitial white blood cell number, dagger (†) indicates significant difference in tubule white blood cell number, Student's *t*-test, one-tailed. (F) Immune complex deposition in the glomerulus of female (NZW×B6.Lbr*^ic^*)F_1_ mice. Frozen kidney sections were stained for the presence of glomerular IgG with Cy3-conjugated sheep anti-mouse IgG, and nuclei counter-stained with DAPI. The corresponding staining of a kidney section from a female (NZW×B6)F_1_ mouse is presented in Fig. S1. Image magnification, 400×.

To evaluate whether a disruption in nuclear structure can contribute to the development of lupus autoimmunity, lupus-prone NZW mice were crossed with non-autoimmune-prone c57Bl/6 mice that are heterozygous for a mutation in *Lbr* that causes an impairment in mRNA splicing (B6.Lbr*^ic^*^/+^ mice). Because the Lbr*^ic^* mutation induces an autosomal dominant disruption in nuclear structure (Hoffmann et al., 2002; Shultz et al., 2003), and a hemizygous NZW background is sufficient to promote autoimmunity, the (NZW×B6.Lbr*^ic^*)F_1_ offspring tested whether the combination of an alteration in nuclear structure and a lupus-prone genetic background results in autoimmunity. Female (NZW×B6.Lbr*^ic^*)F_1_ mice displayed marked splenomegaly at 9 months of age relative to their (NZW×B6)F_1_ littermates (Fig. 2A), but the spleen size of the male (NZW×B6.Lbr*^ic^*)F_1_ and (NZW×B6)F_1_ mice did not differ (Fig. 2B). This is in contrast to the gradual reduction in spleen size in B6 mice with impaired *Lbr* expression (Verhagen et al., 2012). Whereas complete loss of *Lbr* expression causes runting (Shultz et al., 2003), the body weight of (NZW×B6.Lbr*^ic^*)F_1_ mice did not differ from (NZW×B6)F_1_ (Fig. 2B). The development of kidney damage also had a strict female sex bias, with formation of inflammatory foci readily detectable by Masson's Trichrome staining of female (NZW×B6.Lbr*^ic^*)F_1_ kidney sections (Fig. 2C). Female (NZW×B6.Lbr*^ic^*)F_1_ mice had moderate-to-severe glomerulosclerosis and tubulointerstitial fibrosis (Fig. 2D), accompanied by robust perivascular infiltration of mononuclear cells (Fig. 2E). Glomerular deposition of immune complexes was also present in female (NZW×B6.Lbr*^ic^*)F_1_ mice (Fig. 2F), but not in female (NZW×B6)F_1_ littermate controls (Fig. S1), or in F_1_ male mice of either *Lbr* genotype (not shown). Thus, only female (NZW×B6.Lbr*^ic^*)F_1_ mice had splenomegaly and kidney damage – pathological features that are associated with the induction of lupus autoimmunity.

**Fig. 3.**
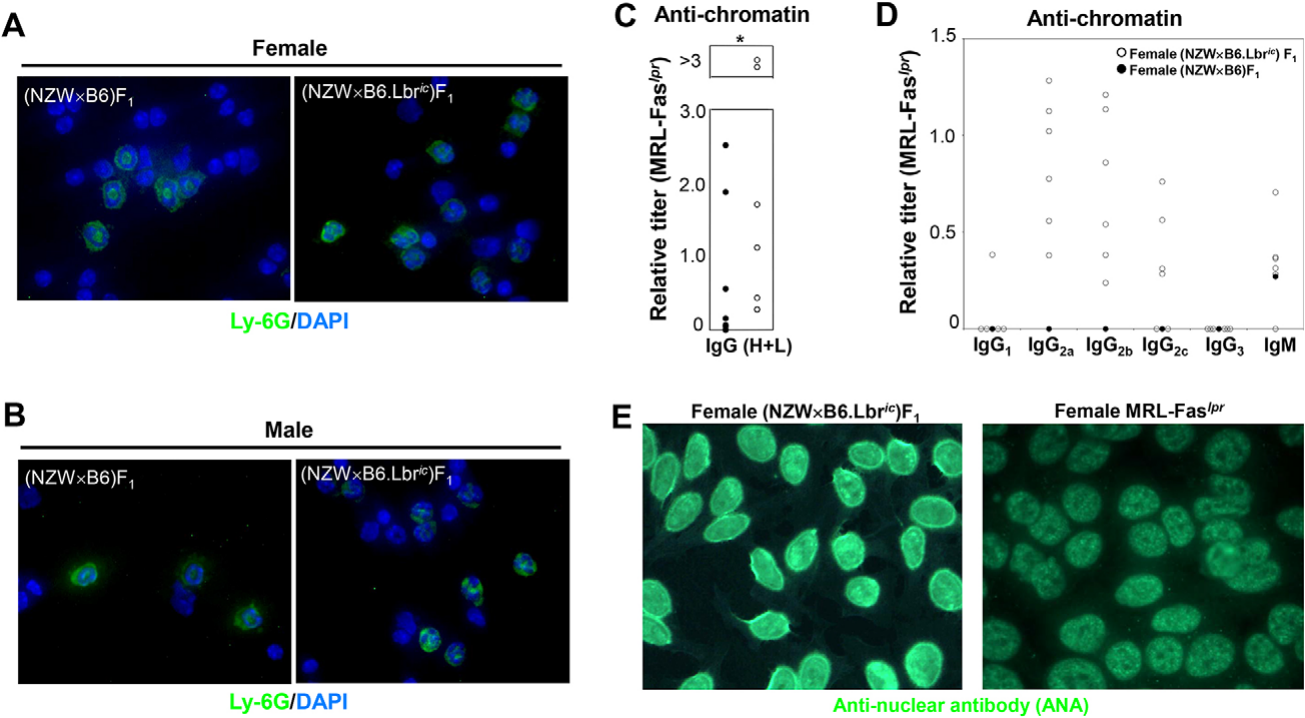
**Development of lupus autoantibodies in female (NZW×B6.Lbr*^ic^*)F_1_ mice.** (A,B) Disruption of neutrophil nuclear morphology in (NZW×B6.Lbr*^ic^*)F_1_ mice. White blood cells isolated from female (A) and male (B) (NZW×B6.Lbr*^ic^*)F_1_ and (NZW×B6)F_1_ mice were stained with FITC-conjugated anti-Ly-6G to identify neutrophils, and the nuclei counter-stained with DAPI. The ring-shaped nuclear morphology of (NZW×B6)F_1_ mice is characteristic of mouse neutrophils, whereas the disorganized nuclear morphology of (NZW×B6.Lbr*^ic^*)F_1_ neutrophils is consistent with the autosomal dominant disruption in *Lbr*. (C) Anti-chromatin ELISA assays of serum from female (NZW×B6)F_1_ (filled circles) and (NZW×B6.Lbr*^ic^*)F_1_ mice (open circles). Serum was screened initially for anti-chromatin IgG titers relative to female MRL-Fas*^lpr^*. (D) Sera from six anti-chromatin IgG-positive (NZW×B6.Lbr*^ic^*)F_1_ mice and one anti-chromatin-negative (NZW×B6)F_1_ mouse were subsequently screened for anti-chromatin titers of IgG_1_, IgG_2a_, IgG_2b_, IgG_2c_, and IgM relative to female MRL-Fas^*lpr*^. (E) Distinct anti-nuclear antibody (ANA) staining patterns of the HEp-2 cell line with sera from female (NZW×B6.Lbr*^ic^*)F_1_ (left panel) and MRL-Fas*^lpr^* mice (right panel). Additional images for female (NZW×B6.Lbr*^ic^*)F_1_ mice, and female (NZW×B6)F_1_ are presented in Fig. S2. Anti-nuclear antibody staining for male mice is presented in Fig. S3. Image magnification, 1000×.

To confirm that the loss of a single copy of *Lbr* was sufficient to induce a disruption in nuclear structure in the (NZW×B6)F_1_ mouse model system, the nuclei of isolated white blood cells were stained with DAPI, and neutrophils were identified by counterstaining with a fluorescently labeled antibody specific for the neutrophil surface marker Ly6G (Fig. 3A,B). The Ly6G-positive cells of (NZW×B6)F_1_ mice of either sex possessed the ring-shaped nuclear morphology that is characteristic of mouse neutrophils (Fig. 3A,B). In marked contrast, the neutrophils of (NZW×B6.Lbr*^ic^*)F_1_ mice displayed prominent alterations in nuclear morphology (Fig. 3A,B), which is consistent with the impairment in *Lbr* expression (Hoffmann et al., 2002; Shultz et al., 2003; Verhagen et al., 2012). The disruption in the nuclear morphology of neutrophils establishes the autosomal dominant alteration in nuclear structure, which is attributable to impaired *Lbr* expression in mice that inherited the Lbr*^ic^* mutation.

Female (NZW×B6.Lbr*^ic^*)F_1_ mice had serum titers of anti-chromatin that exceeded those of aged female MRL-Fas*^lpr^* mice (Fig. 3C). Despite the comparatively high titers of anti-chromatin attained in the female (NZW×B6.Lbr*^ic^*)F_1_ mice, the distribution of anti-double-stranded DNA did not differ from the littermate control female (NZW×B6)F_1_ mice (not shown). Only two female (NZW×B6.Lbr*^ic^*)F_1_ mice, with relative anti-chromatin titers over threefold greater than those attained in the female MRL-Fas*^lpr^* serum standard, developed a detectable titer of anti-double-stranded DNA autoreactivity (not shown). Although titers of anti-chromatin were observed in a few of the female (NZW×B6)F_1_ mice, only one attained a level comparable to MRL-Fas*^lpr^* (Fig. 3C). Male mice did not develop anti-chromatin antibodies (not shown). IgG2a, IgG2b, IgG2c and IgM were prevalent anti-chromatin immunoglobulin subtypes in the (NZW×B6.Lbr*^ic^*)F_1_ sera, and anti-chromatin IgG1 and IgG3 were not detected (Fig. 3D). Similarly, female (NZW×B6.Lbr*^ic^*)F_1_ serum had strong anti-nuclear antibody (ANA) reactivity, displaying a combination of anti-nuclear membrane reactivity and homogenous staining (Fig. 3E; Fig. S2). The nuclear membrane staining profile of female (NZW×B6.Lbr*^ic^*)F_1_ mice was distinct from the purely homogenous pattern of female MRL-Fas*^lpr^* mice (Fig. 3E). Female (NZW×B6)F_1_ littermates displayed little or no anti-nuclear reactivity (Fig. S2), and male mice did not develop anti-nuclear antibodies, irrespective of *Lbr* genotype (Fig. S3). Therefore, the induction of anti-nuclear autoimmunity required an alteration in nuclear structure in a lupus-prone background, and was restricted to female mice.

### Autoantigens recognized by female (NZW×B6.Lbr*^ic^*)F_1_ sera include modified histones

**Fig. 4.**
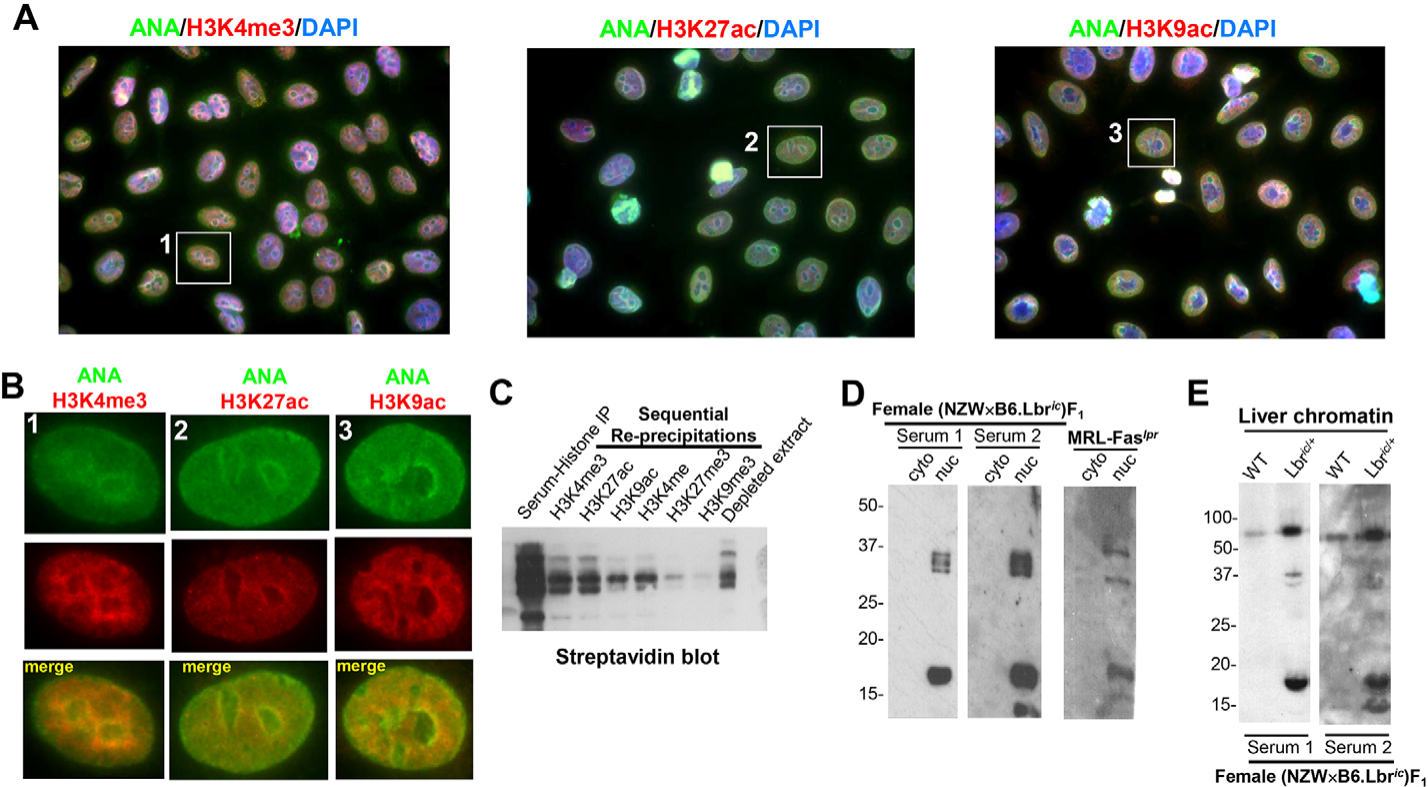
**Female (NZW×B6.Lbr*^ic^*)F_1_ mice develop anti-nuclear autoantibodies recognizing histone H3 modifications associated with gene activation.** (A) Co-localization of anti-nuclear reactivity and the activation-associated histone H3 modifications H3K4me3 (left panel), H3K27ac (middle panel), and H3K9ac (right panel). Anti-nuclear antibody (ANA) staining in the female (NZW×B6.Lbr*^ic^*)F_1_ serum was detected with an Alexa Fluor 488-conjugated goat anti-mouse antibody, anti-modified H3 histones were detected by an Alexa Fluor 594-conjugated goat anti-rabbit secondary antibody. Nuclei were counterstained with DAPI. Image magnification, 1000×. The individual red, green and blue color channels demonstrating the co-localization of the homogenous component of anti-nuclear autoantibody reactivity with modified histones are presented in Fig. S4. (B) Digital enlargement of the individual cells indicated in A. Anti-nuclear staining mediated by the (NZW×B6.Lbr*^ic^*)F_1_ serum is shown in the green channel, and the indicated modified histone is counterstained in the red channel. The yellow overlap signal represents nuclear co-localization of mouse serum immunoreactivity with anti-modified H3 histones. (C) Histones purified from HeLa cells were biotinylated *in vitro*, immunoprecipitated with (NZW×B6.Lbr*^ic^*)F_1_ mouse sera, collected on Protein A/G sepharose beads, washed, and eluted. The contents of the recovered histone extracts, consisting of histones that were specifically recognized by (NZW×B6.Lbr*^ic^*)F_1_ mouse sera, were sequentially re-precipitated with rabbit sera recognizing H3K4me3, H3K27ac, H3K9ac, H3K4me, H3K27me3, H3K9me3. Samples prepared from histone extracts collected from each step before, during, and after the re-precipitation process were blotted using peroxidase-conjugated streptavidin and enhanced chemiluminescence. (D) Cytoplasmic and nuclear extracts prepared from 1×10^6^ B6 mouse embryonic fibroblasts (MEFs) were resolved using a 12% SDS-PAGE gel, and immunoblotted with sera from two female (NZW×B6.Lbr*^ic^*)F_1_ mice, and pooled sera from aged female MRL-Fas*^lpr^* mice. Serum staining from a representative female (NZW×B6)F_1_ mouse, and verification of cytoplasmic and nuclear fractionation, is presented in Fig. S5. (E) Chromatin (5 µg) was isolated from the liver of female wild-type B6 and B6.Lbr*^ic^*^/+^ mice, and immunoblotted with serum from two female (NZW×B6.Lbr*^ic^*)F_1_ mice.

The strong anti-chromatin titer and the homogenous ANA staining component of the female (NZW×B6.Lbr*^ic^*)F_1_ sera suggested anti-histone immunoreactivity. Serum samples from female (NZW×B6.Lbr*^ic^*)F_1_ mice that were previously identified as ANA-positive were subjected to counterstaining with putative nuclear antigens. Because counterstaining human epithelial type 2 (HEp-2) cells for total histones was uninformative, the colocalization of the mouse sera ANA reactivity with specific histone modifications was examined in greater detail (Fig. 4A). Female (NZW×B6.Lbr*^ic^*)F_1_ serum staining colocalized with several histone H3 modifications that are associated with gene expression, including H3K4me3, H3K27ac and H3K9ac (Fig. 4B; Fig. S4). In contrast, histone modifications associated with gene repression, such as H3K27me3 and H3K9me3, did not colocalize (not shown). This selective recognition of activation-associated histone modifications was confirmed by sequential re-precipitation of modified H3 from female (NZW×B6.Lbr*^ic^*)F_1_ serum immunoprecipitates of biotinylated histones (Fig. 4C). Female (NZW×B6.Lbr*^ic^*)F_1_ serum preferentially recovered histone modifications associated with gene activation (Fig. 4C), consistent with the colocalization pattern observed by fluorescence microscopy (Fig. 4A,B; Fig. S4). Female (NZW×B6.Lbr*^ic^*)F_1_ serum immunoblotting of low-molecular-mass proteins of mouse embryonic fibroblast (MEF) cytoplasmic and nuclear extracts confirmed the recognition of histones (Fig. 4D). A prominent band at 16 kDa in the nuclear extracts was also recognized by female MRL-Fas*^lpr^* sera. The nuclear fraction also had bands at 33 kDa, consistent with hnRNP-A2/RA33 (Fig. 4D) (Monneaux et al., 2001). The serum immunoblots using female (NZW×B6)F_1_ serum did not detect any cytosolic or nuclear proteins (Fig. S5). Taken together, these data demonstrate that (NZW×B6.Lbr*^ic^*)F_1_ mice develop an anti-nuclear autoimmune response that selectively recognizes specific modified histones; however, the induction of autoimmunity is restricted to females.

Because nuclear alterations resulting from impaired expression of *Lbr* affect the regulation of gene expression (Solovei et al., 2013) and cell survival (Verhagen et al., 2012), it is possible that the defect in *Lbr* expression generates additional sources of autoantigens due to alterations in chromatin homeostasis. Because the pronounced changes in immune cell number (Fig. 2A,B) and tissue recruitment (Fig. 2D,E) that are present in female (NZW×B6.Lbr*^ic^*)F_1_ mice would obfuscate the analysis of chromatin immunogenicity, a direct examination of the auto-antigenic profile of chromatin isolated from wild-type B6 and B6.Lbr*^ic^*^/+^ was conducted. Chromatin isolated from the liver of female wild-type B6 and B6.Lbr*^ic^*^/+^ mice was immunoblotted with sera from two female (NZW×B6.Lbr*^ic^*)F_1_ mice (Fig. 4E). The serum immunoblots of chromatin isolated from the non-autoimmune-prone wild-type B6 mice displayed only weak and limited immunoreactivity (Fig. 4E). In marked contrast, the B6.Lbr*^ic^*^/+^ liver chromatin had multiple intensely reactive bands, consistent with a robust enhancement of chromatin immunogenicity resulting from impaired *Lbr* expression, even when present in the non-autoimmune-prone B6 genetic background. The profile of induction of serum immunoreactivity to B6.Lbr*^ic^*^/+^ liver chromatin resembled that of nuclear extracts isolated from primary B6 MEF cultures, in which the major epitopes were modified histones at ∼16 kDa and a set of 33-kDa bands that were also detected by MRL-Fas*^lpr^* serum (Fig. 4D). However, unlike the nuclear extracts of MEFs grown *in vitro*, a prominent additional band at 60 kDa was detected in B6.Lbr*^ic^*^/+^ mouse liver chromatin isolated *ex vivo*. These results demonstrate that the disruption of *Lbr* expression induces alterations in chromatin regulation that are sufficient to enhance its potential auto-antigenicity.

### Disruption of Lbr induces autoreactivity to the A-type lamina, but not lamin A/C itself

**Fig. 5.**
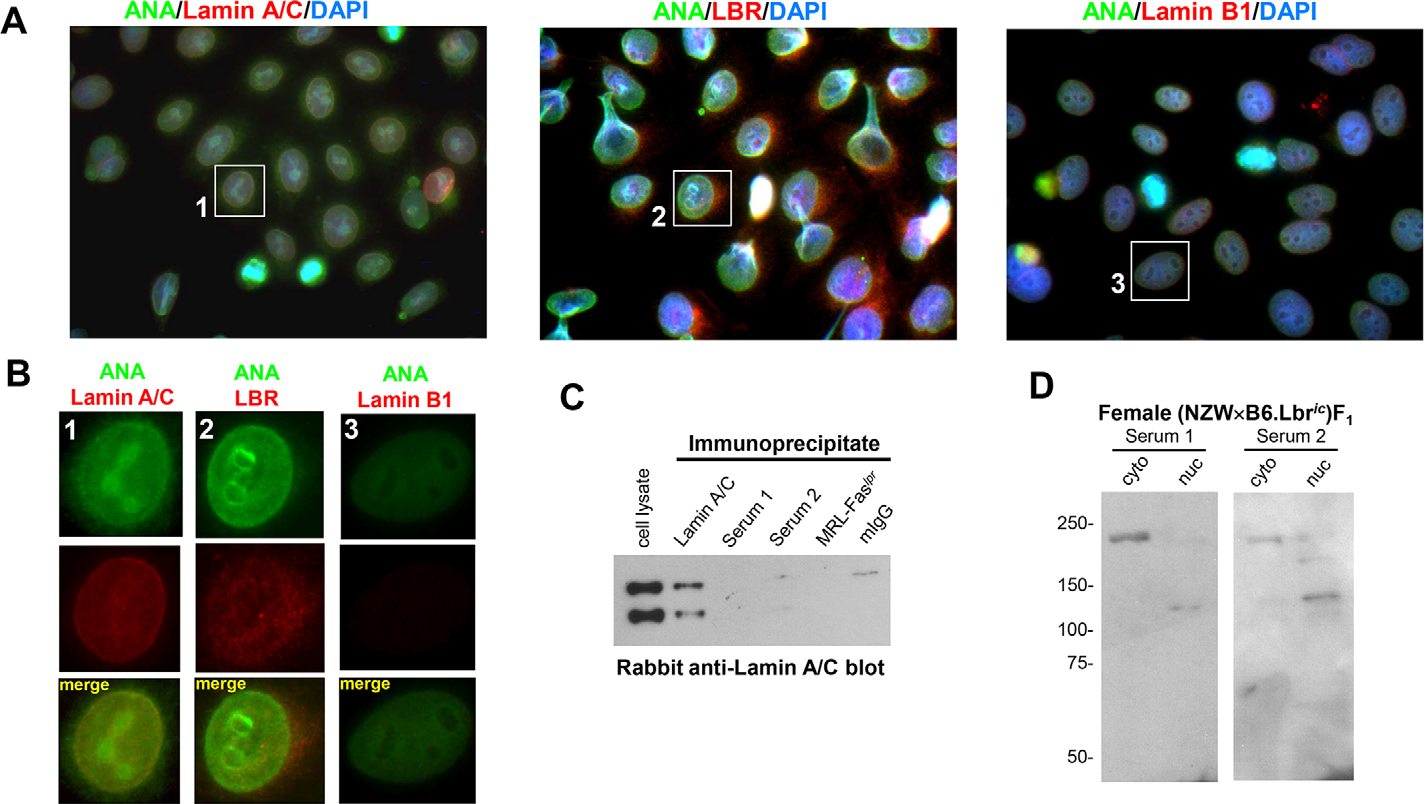
**Female (NZW×B6.Lbr*^ic^*)F_1_ mice develop anti-nuclear autoantibodies recognizing the A-type lamina.** (A) Co-staining of HEp-2 cells with female (NZW×B6.Lbr*^ic^*)F_1_ serum and lamin A/C (left panel), LBR (middle panel), and lamin B1 (right panel). Anti-nuclear antibody (ANA) staining in the serum was detected with an Alexa Fluor 488-conjugated goat anti-mouse antibody, lamina proteins were labeled with the indicated rabbit anti-serum and an Alexa Fluor 594-conjugated goat anti-rabbit secondary antibody. Nuclei were counterstained with DAPI. Image magnification, 1000×. The individual red, green and blue color channels demonstrating the co-localization of the nuclear membrane component of anti-nuclear autoantibody reactivity with the A-type lamina are presented in Fig. S6. (B) Digital enlargement of the individual cells indicated in A. Anti-nuclear antibody staining mediated by the (NZW×B6.Lbr*^ic^*)F_1_ serum is shown in the green channel, and the indicated nuclear envelope protein is counterstained in the red channel. The nuclear membrane colocalization of mouse serum with the anti-lamin A/C is represented by the yellow overlap signal. (C) Lamin A/C immunoblots of 5×10^6^ MEFs immunoprecipitated with a mouse lamin A/C monoclonal antibody, the sera from two female (NZW×B6.Lbr*^ic^*)F_1_ mice (Serum 1 and 2), or pooled aged female MRL-Fas*^lpr^* sera. Non-specific mouse IgG (mIgG) served as a serum control. (D) Cytoplasmic and nuclear extracts prepared from 1×10^6^ MEFs were resolved on a 7.5% SDS-PAGE gel, and immunoblotted with sera from two female (NZW×B6.Lbr*^ic^*)F_1_ mice.

The sera of female (NZW×B6.Lbr*^ic^*)F_1_ mice also displayed anti-nuclear membrane reactivity, which was absent in the MRL-Fas*^lpr^* sera (Fig. 3C). Because components of both the A-type and B-type nuclear lamina are identified targets of autoimmunity (Reeves et al., 1987; Courvalin et al., 1990; Chou and Reeves, 1992; Senecal et al., 1999), the colocalization of the anti-nuclear membrane component of female (NZW×B6.Lbr*^ic^*)F_1_ mouse serum with proteins contained within the lamina was examined (Gotzmann and Foisner, 1999; Holaska et al., 2002). HEp-2 cells were co-stained with ANA-positive female (NZW×B6.Lbr*^ic^*)F_1_ serum and rabbit antibodies recognizing lamin A/C, lamin B1 and LBR (Fig. 5A; Fig. S6). Although all of the nuclear structural proteins were detected in HEp-2 cells, only lamin A/C colocalized with the nuclear membrane reactivity of female (NZW×B6.Lbr*^ic^*)F_1_ serum (Fig. 5A,B; Fig. S6). The recognition of lamin A/C was examined directly by immunoblotting (NZW×B6.Lbr*^ic^*)F_1_ serum immunoprecipitates from MEFs for lamin A/C (Fig. 5C). Lamin A/C was recovered in the control immunoprecipitates, but was not detected in the female (NZW×B6.Lbr*^ic^*)F_1_ mouse serum immunopreciptates, nor in immunoprecipitates from the negative control of MRL-Fas*^lpr^* serum, or non-specific mouse IgG. Likewise, immunoblotting with female (NZW×B6.Lbr*^ic^*)F_1_ sera did not detect lamin A/C immunoprecipitates (not shown). (NZW×B6.Lbr*^ic^*)F_1_ serum immunoblotting of high-molecular-mass proteins of cytoplasmic and nuclear extracts prepared from MEFs revealed a ∼220 kDa band recognized predominantly in the cytoplasmic fraction, and a 120 kDa band in the nuclear fraction (Fig. 5C), confirming the lack of lamin A/C immunoreactivity. No high-molecular-mass proteins were detected in immunoblots using MRL-Fas*^lpr^* serum (not shown), or female (NZW×B6)F_1_ serum (Fig. S5). Thus, in female (NZW×B6.Lbr*^ic^*)F_1_ mice, disrupting the expression of *Lbr* affects the organization of the B-type lamina, but induces the development of autoimmunity directed against components of the A-type lamina, but lamin A/C itself is not the target.

### Anti-neutrophil autoreactivity mediated by IgM is directed against calreticulin, but not MPO or PR3

**Fig. 6.**
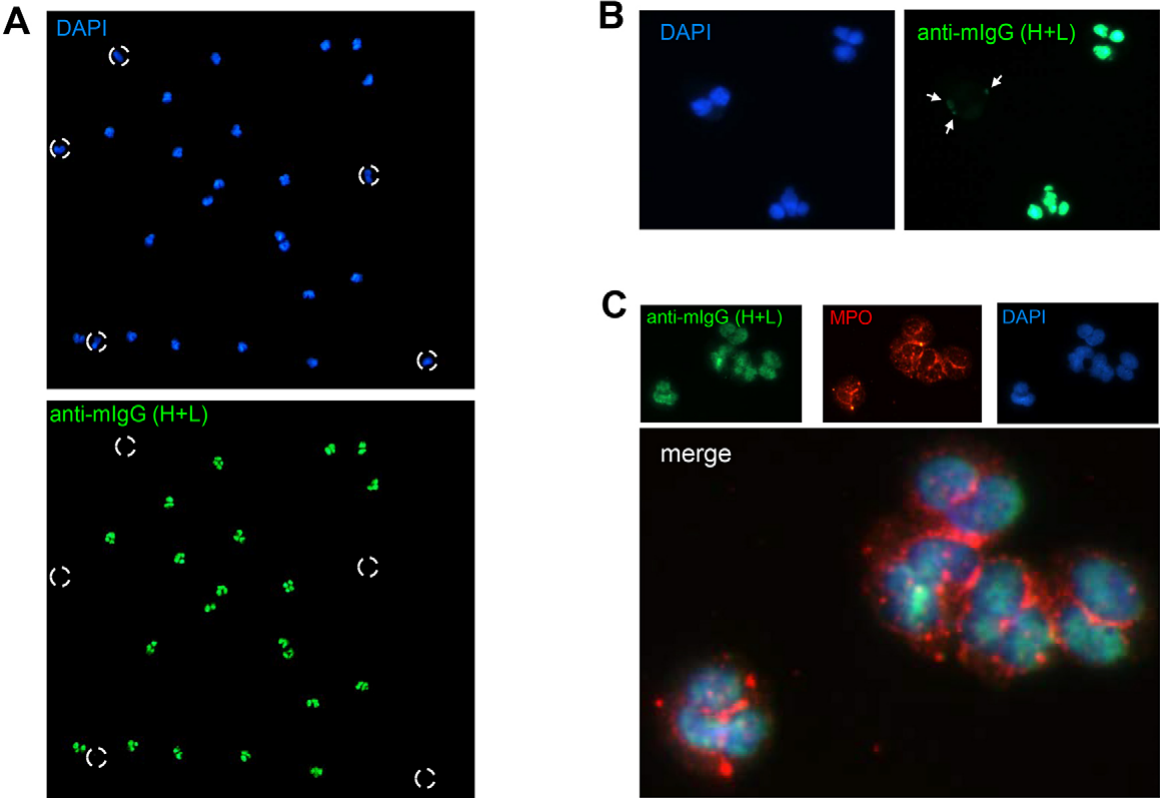
**Anti-neutrophil serum reactivity develops in (NZW×B6.Lbr*^ic^*)F_1_ mice.** (A) Fluorescence microscopy of ethanol-fixed human PMNs stained with DAPI (top panel), and female (NZW×B6.Lbr*^ic^*)F_1_ mouse serum and an Alexa Fluor 488-conjugated goat anti-mouse secondary antibody (bottom panel). Broken circles indicate DAPI-positive bilobed eosinophils that were not readily detected by the female (NZW×B6.Lbr*^ic^*)F_1_ mouse serum. Image magnification, 100×. (B) High-power magnification of ethanol-fixed PMN staining. Neutrophils displayed strong nuclear staining, but eosinophils had comparatively weak and patchy perinuclear staining. Image magnification, 400×. The preferential staining of isolated neutrophils relative to peripheral blood mononuclear cells and isolated monocytes is demonstrated in greater detail in Fig. S7. (C) Ethanol-fixed PMNs were co-stained with female (NZW×B6.Lbr*^ic^*)F_1_ mouse serum (green) and anti-MPO (red), nuclei were counterstained with DAPI (blue). The mouse serum staining did not colocalize with MPO. Image magnification, 1000×.

Because mature hematopoietic cells do not express lamin A/C under resting conditions, and do not have an A-type lamina (Rober et al., 1990; Broers et al., 1997), they would be predicted to lack the anti-nuclear membrane component of (NZW×B6.Lbr*^ic^*)F_1_ serum staining identified in HEp-2 cells. To confirm that the anti-nuclear membrane component in (NZW×B6.Lbr*^ic^*)F_1_ sera recognized the A-type lamina, ethanol-fixed human polymorphonuclear cells were examined by fluorescence microscopy. Quite unexpectedly, female (NZW×B6.Lbr*^ic^*)F_1_ sera displayed robust, and selective, reactivity toward the nuclei of ethanol-fixed neutrophils (Fig. 6A). Segmented mature neutrophils displayed prominent nuclear staining, but the bilobed eosinophils lacked any appreciable staining. (Fig. 6A; Fig. S7). Weak and patchy staining of eosinophils was detected at higher magnification (Fig. 6B), but it was clearly distinct from the intense staining of neutrophils. Variable staining of monocytes within the peripheral blood mononuclear fraction was also observed, but it was also distinct from the prominent staining of isolated neutrophils (Fig. S7). Anti-neutrophil antibodies in autoimmune diseases are defined by their perinuclear (perinuclear anti-neutrophil cytoplasmic antibodies; pANCA) or cytoplasmic (cytoplasmic anti-neutrophil cytoplasmic antibodies; cANCA) staining patterns using ethanol-fixed neutrophils. The strong staining of the neutrophil nuclei in the sera of female (NZW×B6.Lbr*^ic^*)F_1_ mice did not colocalize with myeloperoxidase (MPO; pANCA) or proteinase 3 (PR3; cANCA) (Fig. 6C, and not shown). The lack of anti-MPO and anti-PR3 reactivity was confirmed by ELISA (not shown). Thus, the anti-neutrophil response in female (NZW×B6.Lbr*^ic^*)F_1_ mice is not due to pANCA or cANCA.

**Fig. 7.**
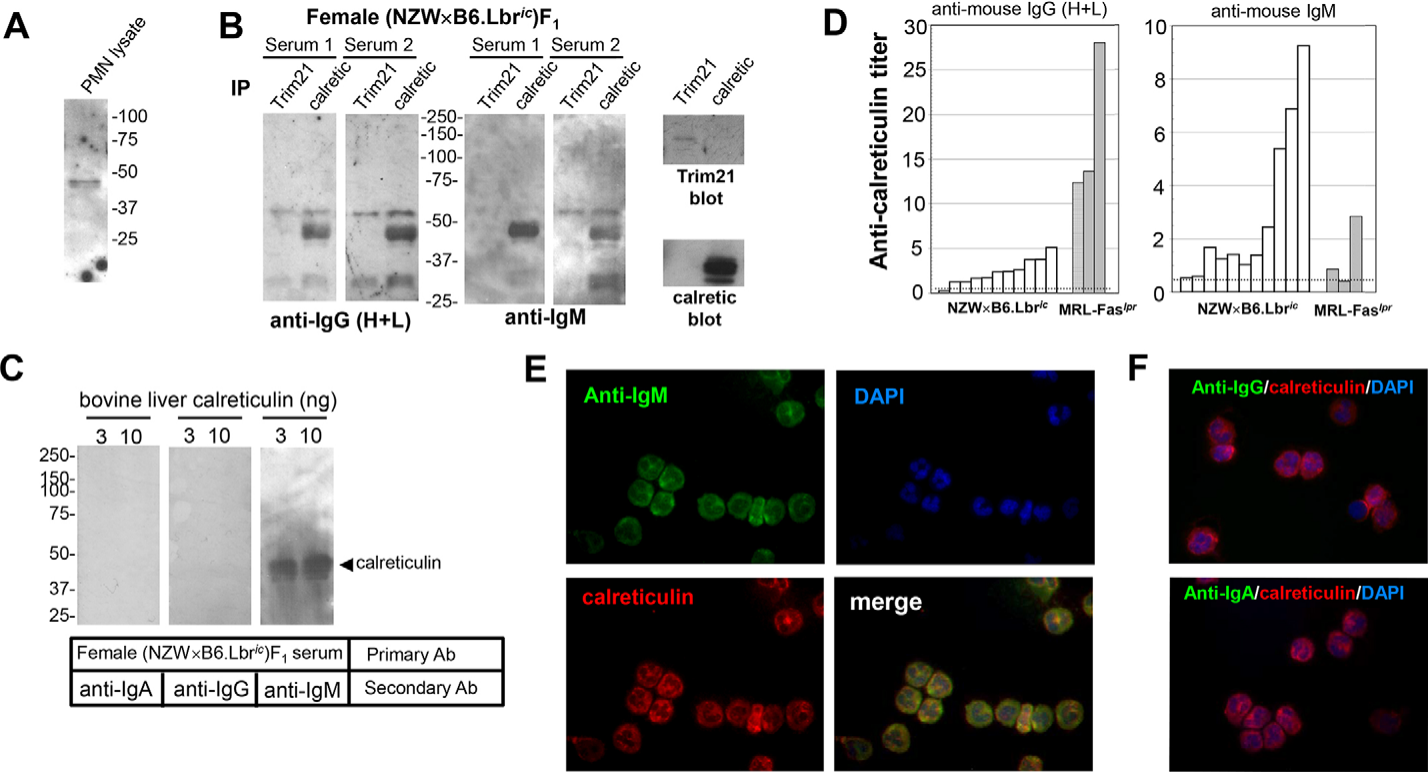
**Anti-calreticulin IgM mediates the anti-neutrophil reactivity in (NZW×B6.Lbr*^ic^*)F_1_ mice.** (A) Triton X-100-soluble extracts of human PMNs were resolved on a 10% SDS-PAGE gel and immunoblotted with female (NZW×B6.Lbr*^ic^*)F_1_ mouse serum. (B) Immunoprecipitates of Trim21 and calreticulin were prepared from 20×10^6^ human PMNs, resolved on a 10% SDS-PAGE gel, serum immunoblotted, and detected with peroxidase-conjugated goat anti-mouse secondary antibodies recognizing IgG (H+L) (left panel) or IgM (right panel). Exposure time for development of the IgM signal was ∼1/10 that of IgG (H+L). Control blots (far right panels) verified recovery of Trim21 and calreticulin in immunoprecipitates. (C) Calreticulin purified from bovine liver was serum immunoblotted and detected with peroxidase-conjugated goat anti-mouse secondary antibodies recognizing IgA (left panel), IgG (middle panel), or IgM (right panel). The location of the calreticulin standard immunoblot using a calreticulin monoclonal antibody is indicated by the arrowhead. (D) IgG (H+L) (left panel) and IgM (right panel) anti-calreticulin ELISA assay of individual female (NZW×B6.Lbr*^ic^*)F_1_ or MRL-Fas*^lpr^* mouse sera. Dashed line represents the mean plus 2 standard deviations from *n*=7 wild-type B6 mice. (E) Paraformaldehyde-fixed PMNs were co-stained with female (NZW×B6.Lbr*^ic^*)F_1_ mouse serum (green) and rabbit anti-calreticulin (red), nuclei were counterstained with DAPI (blue). The co-localization of mouse serum staining and calreticulin was detected using an anti-IgM secondary antibody (merge: yellow signal). Image magnification, 400×. (F) Paraformaldehyde-fixed PMNs were co-stained with female (NZW×B6.Lbr*^ic^*)F_1_ mouse serum (green) and rabbit anti-calreticulin (red), nuclei were counterstained with DAPI (blue). Neither the anti-IgG (top panel), nor anti-IgA (bottom panel) secondary antibodies colocalized the mouse serum staining with calreticulin. Image magnification, 400×.

Female (NZW×B6.Lbr*^ic^*)F_1_ serum immunoblotting of neutrophil lysates identified a protein of ∼48 kDa that was detected by immunoblotting with serum from several mice (Fig. 7A). Because Trim21 and calreticulin are autoantigens within this size range (Kishore et al., 1997; Oke and Wahren-Herlenius, 2012), neutrophil immunoprecipitates of Trim21 and calreticulin were serum-immunoblotted. Immunoblotting with the sera of 6 of 11 female (NZW×B6.Lbr*^ic^*)F_1_ mice detected calreticulin, but Trim21 was not detected (Fig. 7B, and not shown). Although the calreticulin immunoreactivity was apparent, the overall intensity of the immunoblots was weak and fuzzy, suggesting that the secondary antibody provided poor recognition of the mouse primary antibody. To test this possibility, the calreticulin immunoprecipitates were blotted with mouse sera as before, then probed with secondary antibodies specific for IgA, IgG or IgM. The IgM-specific secondary antibody greatly facilitated the detection of calreticulin in the serum immunoblots (Fig. 7B). As before, Trim21was not detected. The IgM specificity of the autoreactive calreticulin antibodies was confirmed by immunoblotting purified calreticulin with female (NZW×B6.Lbr*^ic^*)F_1_ sera and secondary antibodies specific for IgA, IgG, or IgM (Fig. 7C). Sera from female (NZW×B6.Lbr*^ic^*)F_1_ mice, and aged female MRL-Fas*^lpr^* mice, were also examined for autoantibodies recognizing calreticulin by ELISA (Fig. 7D). Using this more sensitive assay system, 9 of the 11 female (NZW×B6.Lbr*^ic^*)F_1_ mice had detectable anti-calreticulin IgM titers, whereas MRL-Fas*^lpr^* mice had predominantly an IgG response (Fig. 7D). The recognition of calreticulin in intact neutrophils was addressed using paraformaldehyde-fixed cells and fluorescence microscopy. The anti-neutrophil reactivity in female (NZW×B6.Lbr*^ic^*)F_1_ sera was detected by an anti-mouse IgM secondary antibody, which clearly co-localized with rabbit anti-calreticulin staining (Fig. 7E). Neither anti-mouse IgA nor IgG yielded a detectable signal (Fig. 7F), confirming results of the serum immunoblots of calreticulin immunoprecipitates (Fig. 7B) and purified calreticulin (Fig. 7C). Thus, female (NZW×B6.Lbr*^ic^*)F_1_ mice developed IgM antibodies recognizing calreticulin.

## DISCUSSION

### Nuclear alterations contribute to the development of lupus autoimmunity

Lupus is characterized by the presence of autoantibodies recognizing the cell nucleus. Considerable research effort has focused on aberrant immune responses in development of the disease (Tsokos, 2011; Choi et al., 2012), and several components of the nuclear lamina are targets of autoimmunity (Lassoued et al., 1988; Chou and Reeves, 1992; Senecal et al., 1999), yet a model proposing that alterations in the structure of the nucleus itself contribute to the development of anti-nuclear autoimmunity remained unexamined. The present study demonstrates that nuclear alterations promote autoimmunity when expressed in conjunction with a lupus-prone genetic background. An autosomal dominant alteration in Lbr splicing, when expressed in the context of a hemizygous lupus-prone NZW genetic background, elicited the induction of several autoantibodies, including anti-modified histones, IgG2 antibodies recognizing chromatin, and IgM directed against calreticulin, as well as moderate-to-severe kidney disease and splenomegaly, that was sex-restricted to only the female F_1_ progeny. This profile of autoantibody development, organ damage, and female sex-bias resembles lupus autoimmunity in humans. Although previous studies reported that impaired expression of Lbr in the B6 background did not promote autoimmunity (Verhagen et al., 2012), combining the impairments in *Lbr* with the lupus-prone NZW genetic background induced robust lupus autoimmunity. Thus, alterations in nuclear structure might not be sufficient to induce autoimmunity in the absence of a lupus-prone genetic background. Since neutrophils from SLE patients display disruptions in *Lbr* splicing and a corresponding Pelger–Huet-like nuclear morphology, similar nuclear alterations might contribute to the development of lupus autoimmunity in individuals with an inherited genetic risk.

### Potential mechanisms for the female sex bias in the development of lupus autoimmunity

The restriction of lupus autoimmunity to female (NZW×B6.Lbr*^ic^*)F_1_ mice mimics human SLE. Current models propose that this female sex bias is related to the influence of steroid hormones on immune system function or cell death pathways (Jog and Caricchio, 2013; Sakiani et al., 2013; Tan et al., 2015; Trigunaite et al., 2015). Female steroid hormones potentiate pro-inflammatory immune responses mediated by, at least in part, estrogen receptor-dependent signaling pathways (Sakiani et al., 2013; Tan et al., 2015), whereas androgens provide anti-inflammatory influences (Trigunaite et al., 2015). In addition, sex-steroid hormones contribute to fundamental differences in the execution of programmed cell death, which results in a predisposition for activation of pro-inflammatory pathways associated with necrosis in females, and immunologically silent apoptotic pathways in males (Jog and Caricchio, 2013). However, the roles for nuclear structure and Lbr in regulating cell responses to sex-steroid hormone receptor activation are not known; likewise, the dysregulation of circulating levels of sex-steroid hormone that are secondary to alterations in nuclear structure mediated by impaired Lbr expression has not been examined.

The genetic model utilized herein cannot exclude the possibility that the Y chromosome encodes genes that reconstitute aspects of Lbr function to prevent the development of lupus autoimmunity in males. Although the Lbr*^ic^* mutation was sufficient to induce an alteration in nuclear morphology in the neutrophils of both male and female (NZW×B6.Lbr*^ic^*)F_1_ mice, only the females developed autoimmunity. This suggests that males can compensate for some, but not all, of the effects of impaired Lbr expression, possibly due to the unique profile of structural proteins that comprise the neutrophil nucleus (Olins and Olins, 2005); or that the mechanisms that mediate the functional redundancy in males are tissue- or cell-selective and not expressed in neutrophils, as reflected by the extensive levels of heterochromatin condensation and gene silencing found in mature neutrophils (Olins and Olins, 2005; Olins et al., 2008).

The Y chromosome encodes over 100 genes, including proteins that bind nucleic acids and regulate nuclear structure (Jangravi et al., 2013), as well as the male-restricted histone demethylases KDM5D and UTY (Wang et al., 1995; Warren et al., 2000; Iwase et al., 2007; Walport et al., 2014). UTY demethylates the repression-associated modified histone H3K27me3 (Walport et al., 2014), whereas KDM5D demethylates the activation-associated modified histone H3K4me3 (Iwase et al., 2007), a prominent target for anti-nuclear autoimmunity in female (NZW×B6.Lbr*^ic^*)F_1_ mice. Thus, an alternative model consistent with the female sex bias in lupus is that the additional genomic complexity provided the Y chromosome allows males to reconstitute defects in nuclear regulation.

### Induction of multiple targets of anti-nuclear autoimmunity in female (NZW×B6.Lbr^*ic*^)F_1_ mice

The ANA staining pattern of female (NZW×B6.Lbr*^ic^*)F_1_ mice represented a combination of anti-nuclear membrane and homogenous nuclear labeling. The homogeneous nuclear staining was attributed to recognition of modified histones, and the reactivity was selective for histones possessing modifications related to gene activation and apoptosis (Liu et al., 2012), including acetylation (van Bavel et al., 2010; Pieterse et al., 2015). It is not yet known whether these histone modifications were detected directly, or if a co-associated mark was recognized. The recognition of acetylated histones might have important ramifications for the therapeutic use of histone deacetylase inhibitors (Pieterse et al., 2015; Tan et al., 2010). Although short-term treatment with histone deacetylase inhibitors might be beneficial as a cancer therapy, prolonged or chronic administration might elicit drug-induced lupus in genetically predisposed individuals (Nambiar et al., 2002; Gottlicher et al., 2001; Bleck and Smith, 1990). Thus, a potential association between epigenetic modulation and the induction of lupus may merit close scrutiny as agents targeting the epigenome are developed and tested in clinical trials.

The anti-nuclear membrane reactivity detected by female (NZW×B6.Lbr*^ic^*)F_1_ serum co-localized with the A-type lamina, but it was not attributable to recognition of lamin A/C itself, indicating that additional nuclear proteins mediate the nuclear membrane staining. Although hematopoietic cells lack an A-type lamina under resting conditions (Rober et al., 1990; Broers et al., 1997), T-lymphocytes transiently re-express components of the A-type lamina upon stimulation (Gonzalez-Granado et al., 2014), suggesting that aberrant reorganization of the nucleus following T-lymphocyte stimulation might facilitate the development of anti-nuclear membrane antibodies.

It is difficult to reconcile the seemingly paradoxical finding of autoreactivity toward the A-type lamina as the impairment in Lbr disrupts the B-type lamina; however, histone modifications also support physical associations with nuclear envelope proteins to maintain nuclear stability (Gotzmann and Foisner, 1999; Polioudaki et al., 2001; Makatsori et al., 2004; Harr et al., 2015). EHMT2/G9a methyltransferase produces H3K9me2, which promotes nuclear stability through an association with lamin A/C (Harr et al., 2015), and is a component of a regulatory complex with Lbr and the heterochromatin-binding protein HP1 (Ye et al., 1997; Polioudaki et al., 2001; Makatsori et al., 2004; Tachibana et al., 2005). H3K9me2 also mediates gene silencing during stem cell differentiation (Wen et al., 2009; Hawkins et al., 2010; Harr et al., 2015) and embryonic development (Tachibana et al., 2002), and reduced levels of H3K9 methylation facilitate production of the activation-associated modified histones H3K9ac (Renneville et al., 2015) and H3K4me3 (Binda et al., 2010), two prominent autoantigens in female (NZW×B6.Lbr*^ic^*)F_1_ mice. In humans, *EMHT2/G9a* is in the well-established lupus-susceptibility interval embedded in the human leukocyte antigen (HLA) locus at 6p21.31 (Brown et al., 2001), and high-resolution fine-mapping of the HLA interval identified a polymorphic variant within a Myb-family binding site in the *EMHT2/G9a* enhancer as a prominent lupus risk allele (Kim et al., 2012; Armstrong et al., 2014). Although rs558702 is in the second intron of the complement protein 2 (*C2*) gene, it is positioned less than 5 kb upstream of the transcriptional start site for *EMHT2*/*G9a*, providing an alternative mechanism for HLA-associated lupus susceptibility through disruption of EMHT2/G9a regulation and epigenetic alterations that affect the A-type lamina.

### Female (NZW×B6.Lbr^*ic*^)F_1_ mice develop an IgM that recognizes calreticulin

In addition to autoantibodies targeting the cell nucleus, there was an unanticipated induction of antibodies recognizing calreticulin. Calreticulin is widely expressed, and is involved in calcium buffering, protein folding, and shuttling between the endoplasmic reticulum and the plasma membrane (Coe and Michalak, 2009). Although it is unclear how impaired expression of Lbr would facilitate the development of IgM autoantibodies recognizing calreticulin, the presence of anti-calreticulin autoantibodies has been described in SLE patients (Kishore et al., 1997; Sanchez et al., 2000), and is associated with increased risk of fetal heart conduction block (Orth et al., 1996; Suzuki et al., 2005). The human *CALR* gene is encoded on chromosome 19, which is prone to genomic alterations in abnormal neutrophils isolated from SLE patients (Singh et al., 2014), and somatic insertion/deletion mutations in exon 9 of calreticulin have been identified in myeloproliferative neoplasms with non-mutated JAK2 kinase (Klampfl et al., 2013; Nangalia et al., 2013; Rumi et al., 2014). A relationship between lupus autoimmunity, myeloproliferative disorders and calreticulin mutations is reminiscent of the incidence of scleroderma in cancer patients with RNA polymerase IIIA mutations (Joseph et al., 2014).

### Environmental factors that mediate nuclear alterations might contribute to lupus autoimmunity

Although the present study used a genetic model to induce nuclear alterations in a lupus-prone genetic background, the relationship between nuclear structure and the development of anti-nuclear autoimmunity suggests a fundamental role for cell biology in the elusive non-genetic components of SLE. Environmental factors proposed to trigger lupus autoimmunity (viral infections, drugs, UV irradiation and cancer) also induce alterations in nuclear structure. The nuclear envelope is a physical barrier for viral entry into the nucleus, and must be breached for successful infection of a host cell (Okada et al., 2005; Kobiler et al., 2012; Porwal et al., 2013), and these virally mediated alterations in nuclear structure coincide with an interferon alpha-driven anti-viral immune response (Elkon and Stone, 2011; Levy et al., 2011; McNab et al., 2015). Similarly, the alterations in nuclear structure that occur in cancer cells might provide a link to autoimmunity (Foster et al., 2010; Hutchison, 2014). Taken together, environmental factors that alter the structure of the nucleus itself might provide crucial contributions to the development of anti-nuclear autoimmunity in individuals with an inherited genetic risk.

## MATERIALS AND METHODS

### Mice

NZW and B6.Lbr*^ic^*^/+^ mice (Jackson Labs) were housed in specific pathogen-free conditions, and handled in accordance with the guidelines of the Temple University IACUC. NZW and B6.Lbr*^ic/+^* mice were bred to generate (NZW×B6.Lbr*^ic^*) and (NZW×B6)F_1_ littermates. To prevent biasing of the experimental results evaluating the incidence of lupus autoimmunity in the F_1_ progeny, all mice were assessed for the development of serum autoantibodies recognizing chromatin and the cell nucleus prior to genotyping. The mouse Lbr*^ic^* allele was genotyped by *Bgl*1 restriction fragment polymorphism analysis (Shultz et al., 2003). The F_1_ progeny were born in predicted ratios, and (NZW×B6.Lbr*^ic^*)F_1_ mice did not display the ichthyosis phenotype of B6.Lbr*^ic/ic^* mice (Shultz et al., 2003).

### Evaluation of glomerulonephritis and kidney damage

Glomerular immune complex deposition was analyzed by fluorescence microscopy (Denny et al., 2006). Pathological evaluation of kidney damage was assessed in paraffin-embedded sections (4 µm) stained with Masson's Trichrome, and conducted in a blinded manner by a nephrologist with experience evaluating mouse kidney sections (D.B.J.). The non-parametric Mann–Whitney test was used for statistical comparisons of tissue damage scores, and the Student's *t*-test compared white blood cell infiltration, with statistical significance of *P*<0.05.

### Assessment of mouse neutrophil nuclear morphology

Approximately 0.1 ml of blood was collected from an isoflurane-anesthetized mouse by retro-orbital bleeding using a glass capillary pipette and transferred to a heparinized microfuge tube. The cells were pelleted by brief centrifugation, and washed twice with an isotonic saline solution. The erythrocytes were lysed in cold ACK solution (Thermo Fisher), and the white blood cells collected by centrifugation. The cell pellets were resuspended in 0.22 μm filtered fetal bovine serum and transferred to a microscope slide using a cytocentrifuge. The spots were fixed in 2% paraformaldehyde at room temperature for 30 min, permeabilized and blocked with 0.1% Triton X-100 with 3% BSA in PBS, and stained with FITC-labeled Ly6G (BioLegend). After washing three times with PBS, the slides were mounted in Prolong Anti-Fade reagent containing DAPI (Life Technologies).

### Characterization of serum anti-nuclear antibodies (ANA)

HEp-2 cells immobilized on microscopy slides (MBL/Bion) were incubated with a 1:200 dilution of mouse sera, and a FITC-conjugated goat anti-mouse immunoglobulin secondary antibody (1:1000; 1010-02, Southern Biotech). ANA slides were counterstained with rabbit antibodies at a dilution of 1:1000 recognizing H3K4me3 (39915), H3K9ac (39137), H3K9me3 (39161), H3K27ac (39133), H3K27me3 (39155), all from Active Motif; Lamin A/C (1:1000; GTX101127, Genetex); Lamin B1 (1:1000; ab16048, Abcam); or Lbr (1:1000; PA5-42709, ThermoFisher Scientific), and DyLight594-conjugated goat anti-rabbit IgG (1:2500; 35561, ThermoFisher Scientific).

### ELISA detection of anti-chromatin, anti-double-stranded DNA, anti-myeloperoxidase (MPO), anti-proteinase 3 (PR3) and anti-calreticulin

Anti-chromatin was detected using chicken erythrocyte chromatin, and anti-double-stranded DNA was detected using calf thymus DNA, with alkaline phosphatase-conjugated goat anti-mouse immunoglobulin secondary antibodies (1:5000; 1032-05, Southern Biotech) (Morris et al., 1990). Serum samples from (NZW×B6.Lbr*^ic^*) and (NZW×B6)F_1_ littermates were diluted 1000-fold, and assayed for anti-chromatin and anti-double-stranded titers normalized to a standard curve constructed from pooled autoimmune sera from aged female MRL-Fas*^lpr^* mice, as previously described (Morris et al., 1990).

Anti-MPO, anti-PR3, or anti-calreticulin were detected by coating 96-well plates with 0.5 µg of MPO or PR3 purified from human granulocytes (Athens Bio), or 0.25 µg of calreticulin purified from bovine liver (Sigma-Aldrich), in carbonate buffer overnight at 4°C. The wells were washed five times with PBS containing 0.05% Tween-20 (PBST), and blocked with 3% BSA in PBST overnight at 4°C. Serum samples were diluted in PBST with 0.5% BSA, incubated overnight at 4°C, washed five times, and incubated for 2 h at room temperature with an alkaline-phosphatase-conjugated goat anti-mouse immunoglobulin, or anti-mouse IgM. The wells were washed five times and developed with p-nitrophenol phosphate solution (Sigma-Aldrich). Relative serum titers were established using mouse monoclonal antibodies recognizing MPO (1:2000; clone 1A1, Abcam), PR3 (1:2000; clone WGM2, Abcam), or calreticulin (1:5000; clone FMC75, Genetex).

### Detection of anti-neutrophil antibodies

Human PMNs were transferred to a microscope slide using a cytocentrifuge, fixed in absolute ethanol at −20°C for 20 min, and blocked in 3% BSA in PBS for 1 h at room temperature. Mouse serum samples were diluted 500-fold in 0.5% BSA in PBS, and co-incubated overnight at 4°C with rabbit antibodies recognizing MPO or PR3. Slides were washed three times with PBS, incubated with Alexa Fluor 488-conjugated goat anti-mouse immunoglobulin (A-11001) and DyLight594-conjugated anti-rabbit immunoglobulin (35561) secondary antibodies (both 1:2000; Life Technologies) with DAPI, coverslipped in Prolong Anti-Fade reagent (Life Technologies), and evaluated for co-localization of the mouse anti-neutrophil antibodies with MPO (Abcam) or PR3 (Santa Cruz). Co-localization with calreticulin used PMNs fixed in 4% paraformaldehyde at room temperature for 30 min and rabbit anti-calreticulin (1:1000; GTX111627, Genetex).

### Western blotting and immunoprecipitation

For identification of calreticulin, extracts of PMNs were prepared in 1% Triton X-100 lysis buffer (1% Triton X-100, 50 mM Tris, 150 mM NaCl, pH 7.5) containing protease and phosphatase inhibitors (Pierce). Detergent-soluble extracts were recovered by centrifugation at 16,000×***g***. Immunoprecipitates were prepared using rabbit antibodies recognizing calreticulin (1:1000; GTX111627, Genetex) or Trim21 (1:100; ab96800, Abcam), collected on Protein A/G beads (BioVision), resolved by SDS-PAGE, transferred to PVDF, and probed with mouse serum and an HRP-conjugated secondary antibody recognizing total murine immunoglobulins (1010-05), IgG (1030-05), IgM (1020-05), or IgA (1040-05) (all 1:2000; Southern Biotech). Immunoprecipitates of lamin A/C were prepared from HeLa lysates in RIPA buffer (Millipore) containing protease and phosphatase inhibitors (Pierce) and rabbit anti-lamin A/C (1:500; GTX101127, Genetex).

Sequential immunoprecipitations were performed on histones isolated from HeLa cells using the Histone Isolation Kit (Active Motif). HeLa cells were cultured in Dulbecco's modified Eagle medium with 10% fetal bovine serum at 37°C in a humidified 5% CO_2_ atmosphere in media containing penicillin and streptomycin. Confluent monolayers of HeLa cells were dislodged by digestion in trypsin-EDTA solution, washed in fresh culture media, and collected by centrifugation. Purified histones (∼5 µg) were diluted in ddH_2_O and labeled with biotin-NHS (Pierce) at room temperature for 1 h before quenching with 1% Triton X-100 lysis buffer. Mouse serum immunoprecipitates were prepared by pre-coating Protein A/G beads with a rabbit anti-mouse bridging antibody. The pre-coated beads were incubated with mouse serum for 2 h at 4°C in 1% Triton X-100 lysis buffer, washed three times, and incubated with the biotinylated histone extracts overnight. After three washes with Triton X-100 buffer, the serum-adsorbed biotinylated histones were eluted with 0.1 ml of 0.5×SDS-PAGE sample buffer and diluted tenfold with 1% Triton X-100 lysis buffer. Sequential re-precipitations were conducted by incubating the biotin-labeled histone extract with a series of affinity-purified rabbit antibodies recognizing specific histone H3 modifications at a final dilution of 1:250 (H3K4me3, H3K27ac, H3K9ac, H3K4me, H3K27me3, H3K9me3; all from Active Motif). Following each round of histone re-precipitation, the cleared biotinylated histone extract was subsequently re-precipitated with a different anti-modified histone antibody. The re-precipitated biotinylated histones were eluted in 2×SDS-PAGE sample buffer, and western blotted using HRP-conjugated streptavidin (Southern Biotech).

### Preparation of nuclear and cytoplasmic extracts

Mouse embryonic fibroblasts (MEFs) were cultured in Dulbecco's modified Eagle medium with 10% fetal bovine serum at 37°C in a humidified 5% CO_2_ atmosphere in media containing penicillin and streptomycin. Confluent monolayers of MEFs were dislodged by digestion in trypsin-EDTA solution, washed in fresh culture media, and collected by centrifugation. MEF cell pellets were washed once in cold hypo-osomotic lysis buffer (10 mM Tris, 10 mM NaCl, 3 mM MgCl_2_, pH 7.5) containing protease and phosphatase inhibitors (Pierce), resuspended in 0.45 ml of the same buffer, incubated at 4°C on a rotator for 15 min, lysed by addition of NP-40 at a final concentration of 0.05%, and incubated again at 4°C on the rotator for 15 min. Lysis was confirmed by Trypan Blue staining, and the nuclei pelleted at 500×***g*** for 10 min. The supernatant was collected as the cytoplasmic fraction and the nuclear pellet was re-extracted, the cytoplasmic fractions were pooled, the pelleted nuclei were extracted into 2×SDS-PAGE sample buffer, and insoluble debris was cleared from each fraction by centrifugation at 21,000×***g***. Fractionation of nuclear proteins was verified by anti-histone H3 immunoblotting using affinity purified rabbit antibodies (1:5000; ab1791, Abcam).

### Isolation of chromatin from mouse liver

Mouse liver chromatin was prepared by a modification of the procedure described by Burlingame and Rubin (1990). The liver of 9-month-old female B6 and B6.Lbr*^ic/+^* mice was homogenized in a blender with five volumes of ice-cold Homogenization buffer (1.2 M sucrose, 3 mM MgCl_2_, 0.6 mM CaCl_2_, 40 mM NaCl, 10 mM sodium acetate pH 6.0, with 200 mM PMSF), and strained through cheesecloth. The homogenate was centrifuged at 750×***g*** for 10 min at 4°C, the supernatant discarded, the pellet resuspended in the original volume of Homogenization buffer supplemented with 0.1% Triton X-100, re-homogenized with a blender, and centrifuged 750×***g*** for 10 min at 4°C. The supernatant was discarded, the recovered isolated nuclei were extracted with two rounds of blender homogenization in 50 ml of ice-cold Buffer A (0.25 M sucrose, 0.25% Triton X-100, 10 mM sodium acetate pH 6.0, with 200 mM PMSF), and collected by centrifugation at 1500×***g*** for 20 min at 4°C. The extracted pellet was washed twice by resuspension and blender homogenization in 50 ml of ice-cold Buffer B (0.25 mM sucrose, 1 mM CaCl_2_, 1 mM Trizma pH 8.0, with 200 mM PMSF), followed by centrifugation at 1500×***g*** for 20 min at 4°C. The pellet of isolated chromatin was resuspended in 1.0 ml of Buffer B, the nucleic acid and protein content was determined by absorbance at 260 nm and 280 nm, respectively, and stored in aliquots at −80°C.

### Human samples

The Institutional Review Boards at Temple University and the University of Michigan approved this study, and subjects gave informed consent in accordance with the Declaration of Helsinki. SLE patients fulfilled the revised American College of Rheumatology criteria (Tan et al., 1982). PMNs and low-density granulocytes (LDGs) were isolated as previously described (Denny et al., 2010; Singh et al., 2014), and nuclear morphology was assessed by Hema3 staining (Thermo Scientific).

Neutrophils from healthy donors were enriched by depletion of eosinophils from the PMN fraction using magnetic bead-assisted cell sorting. Eosinophils were labeled with a biotinylated anti-CD23 antibody (10 ng/ml; clone BU38, Ancell) on ice for 30 min, washed with cold cell isolation buffer (0.5% BSA, 1 mM EDTA, in PBS), and incubated for 30 min with a suspension of anti-biotin paramaganetic microbeads (Miltenyi) on ice, and removed using a magnetic column according to manufacturer's guidelines. Monocytes were isolated from Ficoll-Hypaque preparations of peripheral blood mononuclear cells by incubation with a cocktail of mouse biotinylated monoclonal antibodies recognizing CD3 (clone UCHT1), CD7 (clone 3A1e), CD19 (clone BU12), CD79b (clone SN8/3A2-2E7), CD15 (clone AHN1.1), CD56 (clone ANC7C7), and glycophorin A (clone A63-B/C2) (all supplied by Ancell) on ice for 30 min, washed with cold cell isolation buffer, and labeled with anti-biotin-coated paramagnetic beads (Miltenyi). A magnetic column (Miltenyi) was used to collect the enriched monocyte preparations.

RNA was isolated using TriPure reagent (Roche) and cDNAs were produced using oligo-dT primed reverse-transcription with SuperscriptII (Life Technologies). Human LBR cDNA was amplified using Perfect Taq Polymerase (5-Prime) and the primer pairs: Exon 7 forward GTTGTAGAAGGAACGCCTC; Exon 14 reverse CGGTAGGGCACACGGTGACAGTAC. PCR products were subcloned into pCR2.1 (Life Technologies) for sequencing by dideoxynucleotide termination (GeneWiz). Exon skipping was evaluated by Fisher's exact test, one-tailed.
